# Clozapine Impairs Insulin Action by Up-Regulating Akt Phosphorylation and Ped/Pea-15 Protein Abundance

**DOI:** 10.1002/jcp.22864

**Published:** 2011-05-26

**Authors:** Fabio Panariello, Giuseppe Perruolo, Angela Cassese, Ferdinando Giacco, Ginevra Botta, Alessia PM Barbagallo, Giovanni Muscettola, Francesco Beguinot, Pietro Formisano, Andrea de Bartolomeis

**Affiliations:** 1 Dipartimento di Neuroscienze, Sezione di Psichiatria, Laboratorio di Psichiatria Molecolare, University of Napoli “Federico II”Napoli, Italy; 2 Dipartimento di Biologia e Patologia Cellulare e Molecolare, Istituto di Endocrinologia e Oncologia Sperimentale del CNR, University of Napoli “Federico II”Napoli, Italy

## Abstract

Clinical and experimental evidence indicates that atypical antipsychotics impair glucose metabolism. We investigated whether clozapine may directly affect insulin action by analyzing insulin signaling in vitro and in vivo. Clozapine reduced insulin-stimulated glucose uptake in PC12 and in L6 cells, representative models of neuron and skeletal muscle, respectively. Consistently, clozapine reduced insulin effect on insulin receptor (IR) by 40% and on IR substrate-1 (IRS1) tyrosine phosphorylation by 60%. Insulin-stimulated Akt phosphorylation was also reduced by about 40%. Moreover, insulin-dependent phosphorylation of protein kinase C-ζ (PKC-ζ) was completely blunted in clozapine-treated cells. Interestingly, clozapine treatment was accompanied by an insulin-independent increase of Akt phosphorylation, with no change of IR, IRS1, and PKC-ζ basal phosphorylation. The cellular abundance of Ped/Pea-15, an Akt substrate and inducer of insulin resistance, was also increased following clozapine exposure, both in the absence and in the presence of cyclohexymide, a protein synthesis inhibitor. Similar as in cellular models, in the caudate–putamen and in the tibialis muscle of clozapine-treated C57/BL/KsJ mice, Akt phosphorylation and Ped/Pea-15 protein levels were increased and PKC-ζ phosphorylation was decreased. Thus, in these experimental models, clozapine deranged Akt function and up-regulated Ped/Pea-15, thereby inhibiting insulin stimulation of PKC-ζ and of glucose uptake. J. Cell. Physiol. 227: 1485–1492, 2012. © 2011 Wiley Periodicals, Inc.

Schizophrenia is a major highly debilitating psychiatric disorder with a worldwide prevalence of about 1% (Mueser and McGurk, 2004). Among schizophrenic patients life expectancy is 20% shorter than in general population (Newman and Bland, 1991). This is accounted for, at least in part, by circulatory, respiratory and metabolic illnesses (Dynes, 1969; Felker et al., 1996; Mukherjee et al., 1996; Brown, 1997). Several lines of evidence have indicated that schizophrenic patients have a higher prevalence of impaired glucose tolerance, insulin resistance and type 2 diabetes mellitus than general population (Ryan et al., 2003; Citrome et al., 2005; Thakore, 2005). A family history of type 2 diabetes mellitus is found in 18–19% of schizophrenic patients as compared to 1.2–6.3% in the general population (Mukherjee et al., 1989; Adams and Marano, 1995). A combination of genetic and environmental factors, including lifestyle and medications, is likely to be involved in the dysregulation of glucose metabolism observed in these patients (Citrome and Volavka, 2004; Jin et al., 2004; Lamberti et al., 2004; Newcomer, 2004; Citrome et al., 2007).

Current evidence indicates that first (Perez-Iglesias et al., 2007) and, at a larger extent, second generation antipsychotics (or atypical antipsychotics) are associated with the risk of developing type 2 diabetes mellitus (Wirshing et al., 1998; Sernyak et al., 2002; Mackin et al., 2005; Haupt and Kane, 2007; Perez-Iglesias et al., 2007). Nevertheless, the molecular events underlying their actions are poorly understood.

All antipsychotics share a common mechanism of dopamine D2 receptor occupancy. The D2 receptor family inhibits adenylyl cyclase in downstreaming transductional pathway (Stone and Pilowsky, 2007; Nikam and Awasthi, 2008). In addition, activation of D2 receptor family stimulates the assembly of a complex containing β-arrestin2, protein phosphatase 2 A (PP2A), and Akt (Girault and Greengard, 2004; Beaulieu et al., 2005). Akt, also known as protein kinase B (PKB), is a serine–threonine kinase that has been largely studied for its role in growth factor-mediated cell survival, cell-cycle progression, and transcriptional regulation (Brazil et al., 2004). Akt plays a pivotal role in the regulation of glucose metabolism. It is rapidly activated by insulin and, in skeletal muscle cells and adipocytes, it mediates the translocation of glucose transporter 4 (GLUT4) onto the plasma membrane (Hou and Pessin, 2007). Moreover, Akt also participates in the complex mechanism involved in insulin desensitization (Pirola et al., 2003; Tajmir et al., 2003; Bertacca et al., 2005) and protection from apoptosis (Brazil et al., 2004; Duarte et al., 2008). The molecular mechanisms elicited by Akt are only partially known. Akt phosphorylates a wide variety of substrates, including the antiapoptotic protein Ped/Pea-15 (Trencia et al., 2003).

Ped/Pea-15 is a ubiquitously expressed cytosolic protein which is modulated by phosphorylation at Ser116, by calcium-calmodulin kinase II (CaMKII) and Akt and at Ser104 by PKC (Araujo et al., 1993; Condorelli et al., 1998; Xiao et al., 2002; Trencia et al., 2003; Perfetti et al., 2007). Increased expression of Ped/Pea-15 has been detected in patients with type 2 diabetes and their first-degree relatives (Condorelli et al., 1998; Valentino et al., 2006). Moreover, transgenic mice overexpressing Ped/Pea-15 display abnormal glucose tolerance, insulin resistance and increased susceptibility to develop diabetes following weight gain (Vigliotta et al., 2004). Overexpression of Ped/Pea-15 in cultured skeletal muscle cells inhibits insulin-induced activation of protein kinase C-ζ (PKC-ζ), thereby impairing GLUT4 translocation and glucose uptake (Condorelli et al., 1998, 2001; Vigliotta et al., 2004).

We have hypothesized that PED/PEA-15, which is highly expressed in brain (Sharif et al., 2004) and plays a major role as an apoptotic protein (Fiory et al., 2009), might represent a crucial target for antipsychotics in neurons. On the other end, raised levels of PED/PEA-15 in insulin-sensitive tissues may lead to insulin resistance, which often accompanies the use of such drugs. Therefore, we have used the rat pheochromocytoma PC-12 cells and L6 skeletal muscle cells as a model of brain and peripheral tissues, respectively. We have measured glucose uptake in both cell types as a marker of insulin action. Indeed, both cell types have the ability to uptake glucose and to respond to insulin-mediated signals (Dikic et al., 1994; Dwyer et al., 1999; Leney and Tavaré, 2009). Moreover, whilst glucose uptake has been extensively investigated in muscle cells, recent evidence indicates that insulin may also stimulate similar signaling cascades and translocation of glucose transporters in specific brain areas (Grillo et al., 2009).

Here we show, by in vivo and in vitro paradigms, that clozapine increases Akt activity and Ped/Pea-15 protein accumulation in PC-12 and L6 cells. This is paralleled by decreased PKC-ζ activation and deranged insulin-stimulated glucose uptake.

## Materials and Methods

### General

Media, sera, and antibiotics for cell cultures were from Invitrogen Ltd (Paisley, United Kingdom). Phospho-Ser473 Akt antibodies were purchased from Cell Signaling Technology (Beverly, MA). Actin antibody was from Sigma (St. Louis, MO). Antibodies directed against phospho-PKC-ζ/PKC-ζ, Akt, were from Santa Cruz Biotechnology (Santa Cruz, CA). Insulin receptor substrate-1 (IRS1), IRS2, and phospho-Tyr antibodies were from Upstate Cell Signaling Technology (Lake Placid, NY). Antibodies to Ped/pea-15 have been previously described (Condorelli et al., 2001). Electrophoresis and Western blot reagents were from Bio-Rad (Richmond, VA). 2-Deoxy-[^14^C] glucose and ECL reagents were from GE Healthcare (Piscataway, NJ). Other reagents were from Sigma–Aldrich (St. Louis, MO).

### Cell culture procedures

PC12 transplantable rat pheochromocytoma cells and L6 cells were plated and grown in Dulbecco's modified Eagle's medium (DMEM) containing 10% fetal bovine serum (FBS), 5% horse serum (for PC12 cells), 100 µg/ml streptomycin, and 100 IU/ml penicillin at 37°C in a humidified atmosphere of 95% air and 5% CO_2_.

### Drug preparation

Clozapine was a generous gift of Novartis Pharma (AG, Switzerland). The compound was dissolved in dimethyl sulfoxide at the concentration needed, according to previously described procedures (Kang et al., 2004; Sutton et al., 2007). For in vitro studies, we have incubated PC12 and L6 cells with raising concentrations of clozapine (0.1–25 µM) for different time points (6, 12, 24, and 48 h) in order to choose the best time/concentration combination to adopt in our experimental paradigm. Our results have suggested that the best time/concentration setting in our experimental model was 1.5 µM for 24 h. This choice was mainly determined by the complete absence of cellular toxicity.

### 2-Deoxy-glucose (2-DG) uptake, immunoprecipitation, and Western blotting

2-DG uptake was measured as previously reported (Esposito et al., 2008). Briefly, L6 or PC12 cells were incubated in serum-free Dulbecco's modified Eagle's medium (DMEM) supplemented with 0.2% (w/v) bovine serum albumin for 24 h in the presence or absence of 1.5 µM clozapine. Cells were incubated in glucose-free 20 mM HEPES, pH 7.4, 140 mM NaCl, 2.5 mM MgSO_4_, 5 mM KCl, 1 mM CaCl_2_ (HEPES buffer) and exposed or not to raising insulin concentrations (10–200 nM range) for 30 min. Glucose uptake was measured by incubating cells with 0.15 mM 2-deoxy-d-[14C]glucose (0.5 µCi/assay) for 15 min in HEPES buffer. The reaction was terminated by the addition of 10 µM cytochalasin B, and the cells were washed three times with ice-cold isotonic saline solution before lysis in 1 M NaOH. Incorporated radioactivity was measured in a liquid scintillation counter. For immunoprecipitation experiments, cell lysates were solubilized in lysis buffer containing 50 mM HEPES (pH 7.5), 150 mM NaCl, 10 mM EDTA, 10 mM Na_4_P_2_O_7_, 2 mM sodium orthovanadate, 100 mM NaF, 1 mM phenylmethylsulfonyl fluoride, 10 µg/ml aprotinin, 10 µg/ml leupeptin, pH 7.4, and 1% Triton for 2 h at 4°C. Lysates were clarified by centrifugation at 12,000*g* for 15 min at 4°C, and aliquots were either immunoprecipitated with the indicated antibodies or directly separated by sodium dodecyl sulfate–polyacrylamide gel electrophoresis (SDS–PAGE) before analysis by Western blot, as previously described (Fiory et al., 2004).

### Animal care

Three-month-old male C57/BL/KsJ mice were purchased by the Charles River Laboratories (Milan, Italy). Animals were housed under 12-h light/12-h dark cycle in a temperature and humidity controlled colony room and were given pelleted food and water ad libitum in a specific pathogen-free environment.

### Animal treatment

For in vivo studies, Clozapine was purchased by Sigma–Aldrich (St. Louis, MO). Clozapine was dissolved in lactic acid (0.1% in NaCl), adjusted to pH 5.5 by adding NaOH and administered intraperitoneally to the animals. The dose of clozapine used in these studies was 10 mg/kg for 21 days. This dose was chosen according to previous studies (Cheng et al., 2005), describing that animals treated with 2 mg/kg gained weight more than those treated with 10 mg/kg. In agreement with these findings, we have used 10 mg/kg to limit weight gain-dependent insulin resistance.

### Tissue collection and immunoblotting

Two hours after the last injection the mice were decapitated to obtain protein extracts from cortex and caudate–putamen (CP), using the rat brain atlas by Paxinos and Watson (1997) as an anatomical reference (approximately from bregma 6.70 to 4.20 mm and from bregma 2.20 to −0.40 mm for cortex and CP, respectively). Tissue samples (cortex, CP, and tibialis muscles) were rapidly collected, snap frozen in liquid nitrogen and stored at −80°C for subsequent Western blotting. Tissue samples were homogenized in a Polytron homogenizer (Brinkman Instruments, Westbury, NY) in T-PER TISSUE protein extraction reagent buffer (25 mM bicine, 150 Mm sodium chloride (pH 7.6), 1 mM phenylmethylsulfonyl fluoride, 10 µg/ml aprotinin, 10 µg/ml leupeptin, pH 7.4) (PIERCE, Rockford, IL) according to the manufacturer's instruction. Total homogenates were centrifuged at 12,000*g* for 30 min at 4°C and were subjected to SDS–PAGE with 10% gels. Proteins separated on the gels were electroblotted onto nitrocellulose filters as described above and membranes were probed with specific antibodies, as indicated.

### Data analysis

A one-factor analysis of variance (ANOVA) was used to analyze treatment effect. Any significant ANOVA were further analyzed by Student–Neuman–Keuls post hoc test to determine the specificity of the effect. *P* values <0.05 were considered statistically significant.

## Results

### Effect of clozapine on glucose uptake in cultured cells

**Fig. 1:**
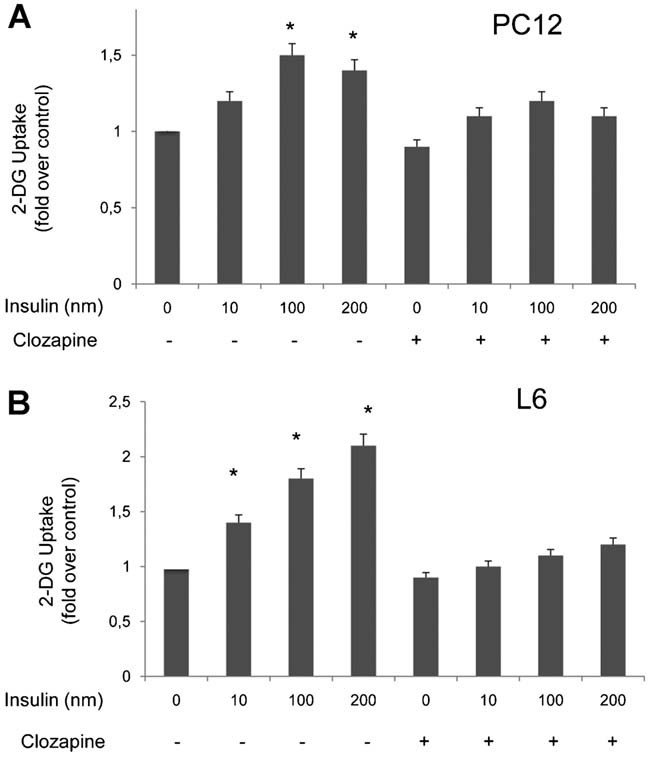
Clozapine inhibits insulin-stimulated glucose uptake in PC12 and L6 cells. 2-Deoxyglucose uptake was measured in PC12 (A) and L6 cells (B) after treatment for 24 h with clozapine (1.5 µM as indicated). Control cells received vehicle (DMSO) instead of drugs. Cells were then exposed to 10, 100, or 200 nM insulin for additional 30 min, as indicated. Bars represent the means ± SD of triplicate determination in four independent experiments. The significant differences (vs. control) were determined by one factor analysis of variance (ANOVA). Positive samples were further analyzed by Student–Neuman–Keuls post hoc test to determine the specificity of the effect. *P < 0.05.

We have evaluated 2-DG uptake in PC12 cells following treatment with 1.5 µM clozapine for 24 h (Fig. 1A). This concentration, although not fully consistent with in vivo dosage range, was chosen in agreement with the results of dose–response and time-course profiles (data not shown). In the absence of insulin, no significant change of 2-DG uptake was induced by clozapine (Fig. 1A). However, insulin effect was almost completely blunted by clozapine. Comparable results were obtained following identical treatment of L6 myotubes (Fig. 1B). Indeed, insulin elicited 2-DG uptake up to 1.5- and 2.1-fold, respectively, in PC12 and in L6 cells, while it failed to induce significant changes in the presence of clozapine (Fig. 1A,B).

### Effect of clozapine on insulin signaling in cultured cells

**Fig. 2:**
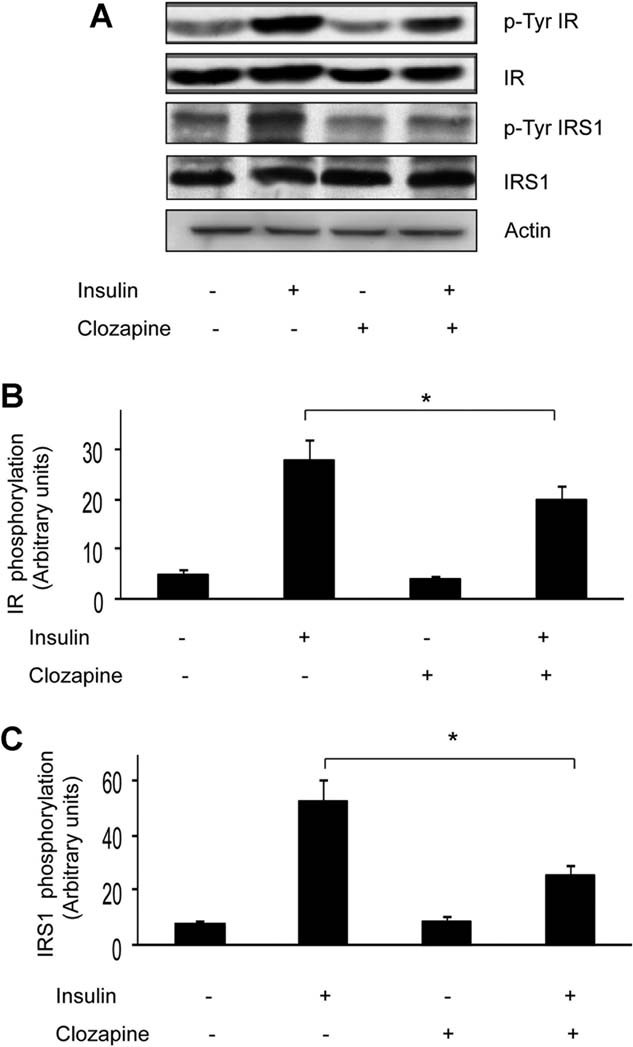
Effect of clozapine on insulin receptor activity in L6 cells. L6 cells have been incubated with clozapine 1.5 µM for 24 h and with insulin 100 nM for further 30 min. A: Protein extracts were immunoprecipitated with anti-IR (α-subunit) and IRS-1 antibodies, subjected to Western blotting with antiphosphotyrosine (p-Tyr), IR (β-subunit), and IRS-1 antibodies, as indicated. Total cell lysates were also blotted with antiactin antibodies to ensure equal amounts of cellular proteins. The blots were revealed by ECL and autoradiography. The blots shown in (A) are representative of four independent experiments. B,C: Bars represent the means ± SD of the ratio of the densitometric values obtained for phospho- and total antibodies. The significant differences, were determined by ANOVA. Positive samples were further analyzed by Student–Neuman–Keuls post hoc test to determine the specificity of the effect. *P < 0.05.

**Fig. 3:**
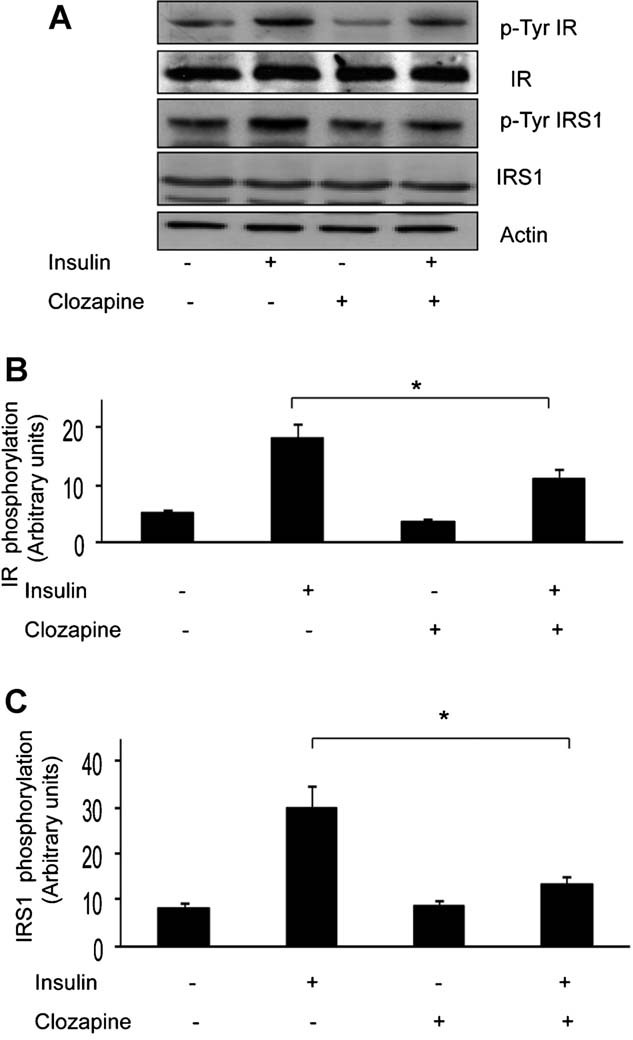
Effect of clozapine on insulin receptor activity in PC12 cells. PC12 cells have been incubated with clozapine 1.5 µM for 24 h and with insulin 100 nM for further 30 min. A: Protein extracts were immunoprecipitated with anti-IR (α-subunit) and IRS-1 antibodies, subjected to Western blotting with antiphosphotyrosine (p-Tyr), IR (β-subunit), and IRS-1 antibodies, as indicated. Total cell lysates were also blotted with antiactin antibodies to ensure equal amounts of cellular proteins. The blots were revealed by ECL and autoradiography. The blots shown in (A) are representative of four independent experiments. B,C: Bars represent the means ± SD of the ratio of the densitometric values obtained for phospho- and total antibodies. The significant differences, were determined by ANOVA. Positive samples were further analyzed by Student–Neuman–Keuls post hoc test to determine the specificity of the effect. *P < 0.05.

In order to investigate at what level insulin action was impaired, L6 cells have been incubated with 1.5 µM clozapine for 24 h and with 100 nM insulin for additional 30 min. Concentration and time were chosen according to the maximal effect obtained in previous experiments. No significant change of the cellular content of the insulin receptor (IR) and of the IRS1 was detected by Western blot with specific antibodies. However, clozapine-treated cells displayed a reduction of insulin-stimulated IR (Fig. 2A,B) and IRS1 (Fig. 2A,C) tyrosine phosphorylation of about 40% and 60%, respectively. Comparable results were obtained by stimulating PC12 cells with insulin (Fig. 3).

**Fig. 4:**
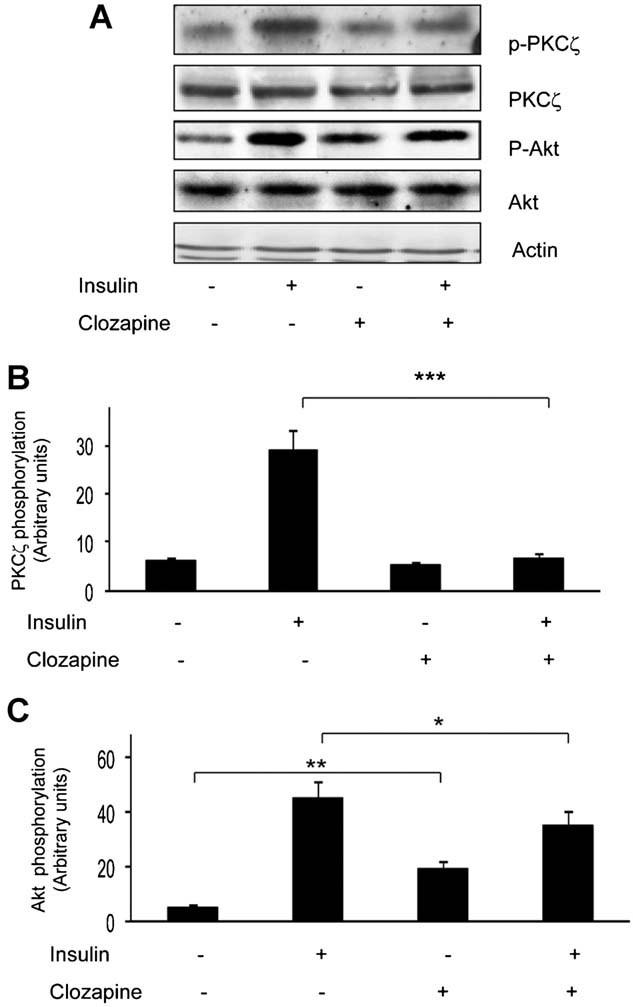
Effect of clozapine on insulin signaling in L6 cells. L6 cells have been incubated with clozapine 1.5 µM for 24 h and with insulin 100 nM for further 30 min. Cell lysates were blotted with phospho-Ser-473 Akt (p-Akt) or phospho-Thr 410 PKC-ζ (p-PKC-ζ) followed by reblotting with total Akt, PKC-ζ, and actin antibodies, as indicated. The blots were revealed by ECL and autoradiography. The blots shown in (A) are representative of four independent experiments. B,C: Bars represent the means ± SD of the ratio of the densitometric values obtained for phospho- and total antibodies. The significant differences, were determined by ANOVA. Positive samples were further analyzed by Student–Neuman–Keuls post hoc test to determine the specificity of the effect. *P < 0.05, **P < 0.01, ***P < 0.001.

**Fig. 5:**
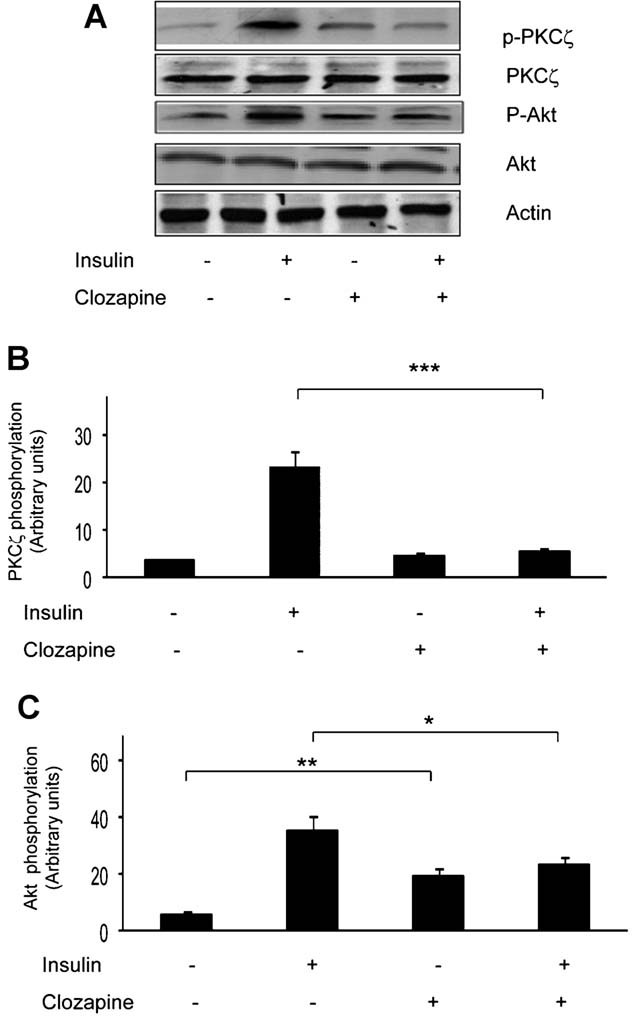
Effect of clozapine on insulin signaling in PC12 cells. PC12 cells have been incubated with clozapine 1.5 µM for 24 h and with insulin 100 nM for further 30 min. Cell lysates were blotted with phospho-Ser-473 Akt (p-Akt) or phospho-Thr 410 PKC-ζ (p-PKC-ζ) followed by reblotting with total Akt, PKC-ζ, and actin antibodies, as indicated. The blots were revealed by ECL and autoradiography. The blots shown in (A) are representative of four independent experiments. B,C: Bars represent the means ± SD of the ratio of the densitometric values obtained for phospho- and total antibodies. The significant differences, were determined by ANOVA. Positive samples were further analyzed by Student–Neuman–Keuls post hoc test to determine the specificity of the effect. *P < 0.05, **P < 0.01, ***P < 0.001.

We also investigated whether PKC-ζ and Akt, two major kinases involved in the regulation of glucose uptake, were affected by clozapine. Protein levels of PKC-ζ were similar in untreated cells and in L6 cells exposed to clozapine, while insulin-induced PKC-ζ phosphorylation at Thr 410 was almost abolished (Fig. 4A,B). No significant change in basal PKC-ζ phosphorylation was detected, instead. Akt cellular content was also unchanged by clozapine (Fig. 4A). Nevertheless, insulin-independent phosphorylation at Ser 473 (which represents an activation marker) was significantly increased by about threefold (Fig. 4A,C). In parallel with the early signaling steps, insulin effect on Akt phosphorylation was reduced by 40% upon clozapine pre-treatment of the cells. Very consistent results were obtained in PC12 cells (Fig. 5).

### Effect of clozapine on Ped/Pea-15 expression

**Fig. 6:**
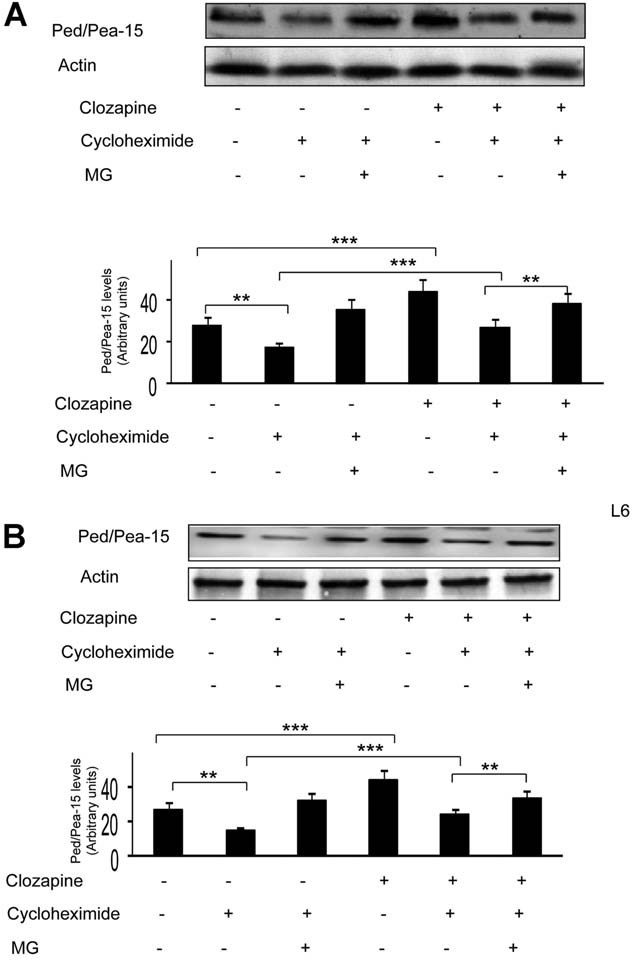
Clozapine increases Ped/Pea-15 protein abundance. L6 (A) and PC12 (B) cells have been treated with clozapine 1.5 µM for 24 h with or without cycloheximide 30 µM (CHX) and MG132 10 µM (MG), as indicated. Cells were lysed as described in the Materials and Methods Section and lysates were blotted with anti-Ped/Pea-15 antibody. Equal loading of the samples was ensured by control blot with antiactin antibodies. The blots were revealed by ECL and autoradiography and subjected to densitometric analysis as described in the Materials and Methods Section. The blots shown are representative of four independent experiments. The significant differences (vs. control) were determined by ANOVA. Student–Neuman–Keuls post hoc test was used to determine specificity of the effect. **P < 0.01, ***P < 0.001.

Next, we addressed whether clozapine may affect the cellular content of Ped/Pea-15, an Akt substrate (Trencia et al., 2003), whose overexpression causes insulin resistance in cellular and animal models (Condorelli et al., 1998; Vigliotta et al., 2004) and associates to insulin resistance in humans (Valentino et al., 2006). Clozapine treatment of L6 cells (Fig. 6A) for 24 h increased Ped/Pea-15 cellular levels by about twofold. Despite a reduction in the absolute amount, raised Ped/Pea-15 content was also observed in the presence of cycloheximide (30 µM), a protein synthesis inhibitor, suggesting that, at least in part, regulation occurred at post-translational level. In this condition, no significant evidence of toxicity has been observed (data not shown). Interestingly, treatment of the cells with 10 µM MG132, rescued the reduction of Ped/Pea-15 levels observed in the presence of cycloheximide, suggesting that most of protein loss occurred via proteasomal degradation. At variance, actin levels remained stable, according to the longer half-life of the protein. Again similar results were obtained with PC12 cells (Fig. 6B).

**Fig. 7:**
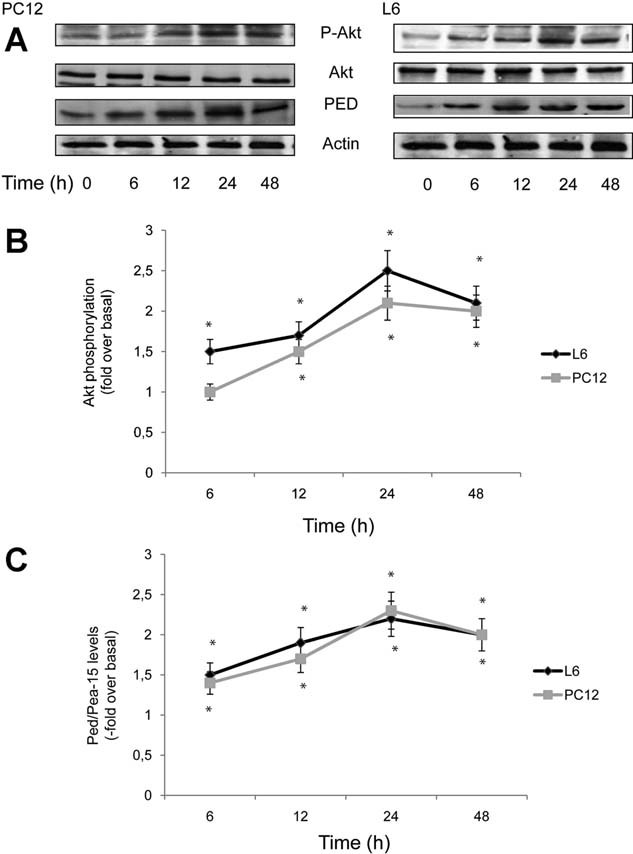
Time-course of clozapine effect on Ped/Pea-15 and Akt in PC12 and L6 cells. The PC12 and L6 cells were incubated for different times with increasing concentrations 1.5 µM clozapine for 6, 12, 24, and 48 h, as indicated. The cells were lysed as described, subjected to Western blotting with phospho-Ser-473 Akt (pAkt) and Ped/Pea-15 and reblotting for total. Equal loading of the samples was evaluated by control blot with antiactin antibodies. The blots were revealed by ECL and autoradiography (A). For phosphorylated Akt, values were expressed as ratio with total Akt levels (B); for Ped/Pea-15 as arbitrary units derived from densitometric analysis, further normalized on actin values (C), and shown with the bars. Data represent the means ± SD from at least three experiments. ANOVA and Student–Neumans–Keuls post hoc test analysis revealed significant differences from the controls. *P < 0.05.

Therefore, PC12 and L6 cells were incubated with 1.5 µM clozapine for different time points (6, 12, 24, and 48 h), prior to measure the levels of phosphorylated Akt and Ped/Pea-15. The time-dependent increase of phosphorylated Akt (Fig. 7A,B) closely paralleled that of Ped/Pea-15 cellular content (Fig. 7A,C), in both PC12 and L6 cells.

### In vivo effects of clozapine on Akt, Ped/Pea-15, and PKC-ζ

In order to verify whether clozapine may induce comparable changes in vivo, two groups of eight C57/BL/KsJ mice were treated with clozapine (10 mg/kg) or vehicle.

**Fig. 8:**
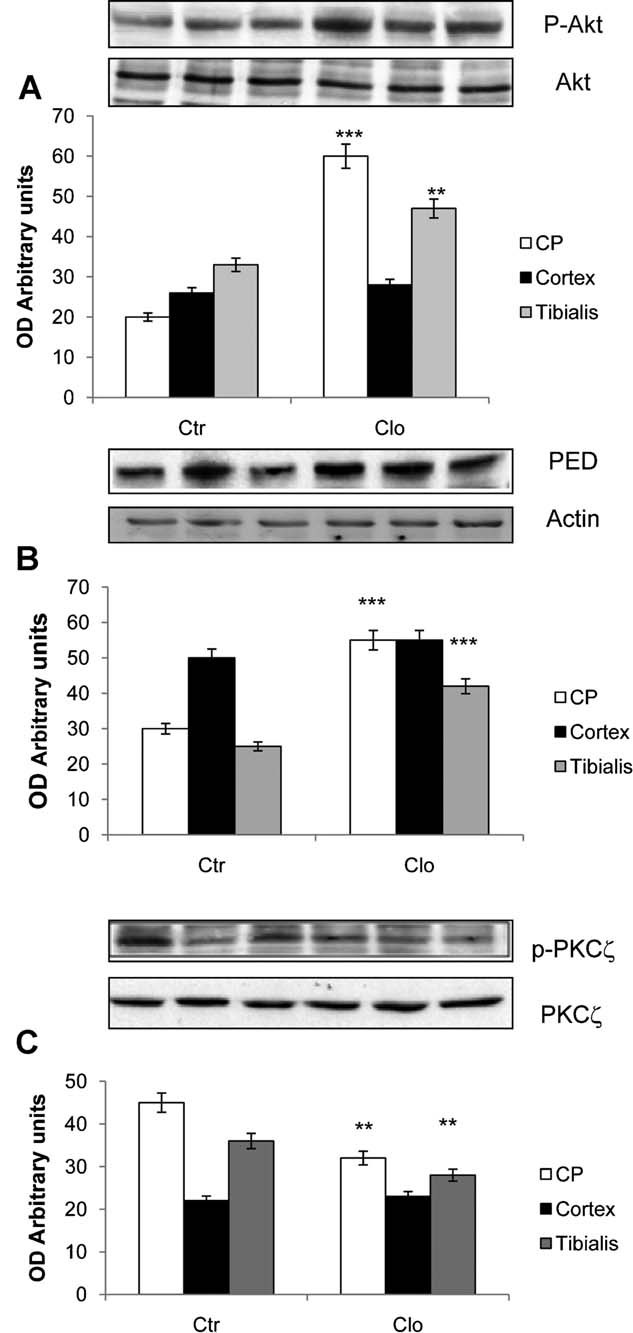
Clozapine effect in caudate–putamen, cortex, and tibialis muscle. Two groups of eight mice were treated once a day for 21 days with single injection of saline, and clozapine (10 mg/kg). A: Phospho-Ser-473 Akt (pAkt), (B) Ped/Pea-15, and (C) phospho-PKC-ζ (p-PKC-ζ) levels were measured in caudate–putamen (CP), cortex, and tibialis muscle, 30 min after the last injection of clozapine or saline. Equal loading of the samples was ensured by control blot with antiactin antibodies. Phosphorylated Akt and PKC-ζ were expressed as ratio with total Akt and total PKC-ζ levels, Ped/Pea-15 as arbitrary units derived from densitometric analysis. All values were further normalized on actin values. Significant differences compared to the respective saline-treated control have been indicated by asterisks, **P < 0.01, ***P < 0.001.

Protein extracts from CP and cortex were obtained and assayed by Western blot with specific antibodies. In the animals treated with clozapine, increased levels of phosphorylated Akt were detected in CP (Fig. 8A), but not in cortex. This was paralleled by increased Ped/Pea-15 detection (Fig. 8B) and reduced PKC-ζ phosphorylation in CP (Fig. 8C). Again, no difference was detected in cortex of the treated animals.

Similarly, tibialis muscle specimens from clozapine-treated mice featured increased phosphorylated Akt (Fig. 8A) and Ped/Pea-15 levels (Fig. 8B) as well as decreased PKC-ζ phosphorylation (Fig. 8C), compared to vehicle-treated controls.

## Discussion

Consistent with previous findings in adipocytes (Vestri et al., 2007), exposure of PC12 and L6 cells, representative models of neuron and skeletal muscle cell, respectively, to clozapine caused an inhibition of insulin-stimulated glucose uptake.

Clozapine, impaired the early events of insulin signaling, inhibiting IR tyrosine auto-phosphorylation and kinase activity and insulin-stimulated Akt phosphorylation. In the absence of insulin, phosphorylated Akt was increased following treatment with clozapine. The increased Akt phosphorylation is consistent with previous observations suggesting that this protein represents a potential target of antipsychotic drugs (Kang et al., 2004; Lu and Dwyer, 2005). In addition, Akt has been envisioned as one of the potential schizophrenia susceptibility genes (Emamian et al., 2004; Lovestone et al., 2007). Thus, one might speculate, that clozapine could operate by enhancing Akt function, thereby counteracting the defect in schizophrenic patients.

However, whether the increase of Akt phosphorylation induced by clozapine might also be involved in the impairment of insulin action on glucose uptake is less intuitive. Indeed, Akt is rapidly stimulated by insulin and mediates many insulin signals, including glucose uptake (Brazil et al., 2004; Hou and Pessin, 2007). On the other hand, Akt is also involved in insulin desensitization (Pirola et al., 2003; Tajmir et al., 2003; Bertacca et al., 2005), and increased Akt has been detected in several models of insulin resistance (Pirola et al., 2003; Tajmir et al., 2003; Bertacca et al., 2005; Chakraborty, 2006). Thus, it could be hypothesized that long-term treatment with clozapine may induce persistent up-regulation of Akt, which in turn may impair insulin signaling.

One of the potential mechanisms by which Akt may affect insulin signaling is the increase of Ped/Pea-15 protein stability. Indeed, Ped/Pea-15 is phosphorylated by Akt at serine 116 (Trencia et al., 2003). This stabilizes the protein, likely preventing its degradation in the proteasome (Trencia et al., 2003; Perfetti et al., 2007). Increased expression of Ped/Pea-15 has been associated to type 2 diabetes in humans (Condorelli et al., 1998; Valentino et al., 2006). Moreover, forced expression in mice and in cultured cells inhibits insulin action on glucose uptake by impairing PKC-ζ activation (Condorelli et al., 2001; Vigliotta et al., 2004). Interestingly, insulin-stimulated PKC-ζ phosphorylation is strongly inhibited in clozapine-treated cells, in parallel with the dramatic inhibition of glucose uptake.

Ped/Pea-15 is also involved in the regulation of apoptotic cell death. Different experimental findings have suggested that over-expression of Ped/Pea-15 protects against stress-induced apoptosis (Trencia et al., 2003; Perfetti et al., 2007; Eckert et al., 2008). Thus, it is conceivable that elevated Ped/Pea-15 levels due to Akt activation, may contribute to the antiapoptotic action of antipsychotics in specific subsets of neurons. On the other hand, raised Ped/Pea-15 levels may contribute as well to the diabetogenic potential of such compounds, impairing insulin action in peripheral tissues.

In vivo, increased Akt phosphorylation and Ped/Pea-15 expression and decreased PKC-ζ phosphorylation have been detected in the CP and in the tibialis muscles of clozapine-treated mice, suggesting that the skeletal muscle may represent a direct target for antipsychotics. Overall, the finding that clozapine, decreases the early steps of insulin action, that is, receptor and IRS phosphorylation, is consistent with the impact exerted by atypical antipsychotics on glucose tolerance (Wirshing et al., 1998). Our findings are also consistent with previous observations (Vestri et al., 2007) showing that atypical antipsychotics directly modulate insulin action in insulin responsive tissues. Moreover, the rapid onset of hyperglycemia in clozapine- or olanzapine-treated patients, without weight gain, and the restoration of normal glucose metabolism after antipsychotic discontinuation, support the relevance of direct peripheral action of antipsychotics and their modulation of glucose metabolism (Koller et al., 2001; Koller and Doraiswamy, 2002). In addition, it has been shown that olanzapine causes impairment of β cell function and insulin resistance in dogs (Ader et al., 2005). Another possibility, is that derangement of glucose metabolism accompanying the treatment with antipsychotics is due to their action at the level of central nervous system. In this regard it should be noticed that serotonin system, which is also affected by atypical antipsychotics, is deeply involved in feeding behavior. Indeed, animal knockout model of 5-hydroxytryptamine-2C receptor (5-HT2c) display obesity and altered feeding behavior (Lebovitz, 2003; Reynolds, 2004). Moreover increased food intake and weight gain observed under clozapine treatment can be reversed by D1, D2, 5-HT1b, 5-HT2c, and 5-HT3 agonists, arguing that dopaminergic and serotoninergic receptors are involved in antipsychotic-induced weight gain, insulin resistance, and diabetes (Kaur and Kulkarni, 2002).

Although current literature does not exclude central effects of antipsychotics in causing impairment in peripheral glucose metabolism (Lean and Pajonk, 2003; Howes et al., 2004), our data support the hypothesis that clozapine may directly act on peripheral tissues, affecting insulin sensitivity.

The mechanisms responsible for clozapine effects are currently under investigation in our laboratory, and may also involve the increases of Akt. Indeed, it has been previously reported that Akt hyper-activation impairs IRS1 function, and may affect IR activity as well (Brozinick et al., 2003). One might speculate that a different subcellular localization of Akt following exposure to clozapine may affect its function toward specific substrates rather than others.

In summary, our data suggest that second generation antipsychotics may contribute to the impairment of glucose metabolism by interfering with insulin action. Drug-induced deregulation of Akt enhances the levels of Ped/Pea-15, which, in turn, inhibits the activation of atypical PKC-ζ and prevents insulin-stimulated glucose uptake.
